# DArTseq genotyping facilitates the transfer of “exotic” chromatin from a *Secale cereale* × *S. strictum* hybrid into wheat

**DOI:** 10.3389/fpls.2024.1407840

**Published:** 2024-09-06

**Authors:** Kitti Szőke-Pázsi, Klaudia Kruppa, Zuzana Tulpová, Balázs Kalapos, Edina Türkösi, Eszter Gaál, Éva Darkó, Mahmoud Said, András Farkas, Péter Kovács, László Ivanizs, Jaroslav Doležel, M. Timothy Rabanus-Wallace, István Molnár, Éva Szakács

**Affiliations:** ^1^ Department of Biological Resources, Agricultural Institute, HUN-REN Centre for Agricultural Research, Martonvásár, Hungary; ^2^ Institute of Experimental Botany, Centre of Plant Structural and Functional Genomics, Olomouc, Czechia; ^3^ Field Crops Research Institute, Agricultural Research Centre, Giza, Cairo, Egypt; ^4^ School of Agriculture, Food, and Ecosystem Sciences, The University of Melbourne, Melbourne, VIC, Australia; ^5^ Research Group Genomics of Genetic Resources, Leibniz Institute of Plant Genetics and Crop Plant Research (IPK), Gatersleben, Germany

**Keywords:** *Secale cereanum*, *Triticum aestivum*, DArTseq markers, genotyping, heatmap, introgression lines, chromosome rearrangements

## Abstract

Cultivated and wild species of the genus rye (*Secale*) are important but underexploited gene sources for increasing the genetic diversity of bread wheat. Gene transfer is possible via bridge genetic materials derived from intergeneric hybrids. During this process, it is essential to precisely identify the rye chromatin in the wheat genetic background. In the present study, backcross generation BC_2_F_8_ from a cross between *Triticum aestivum* (Mv9kr1) and *S. cereanum* (‘Kriszta,’ a cultivar from the artificial hybrid of *S. cereale* and *S. strictum*) was screened using *in-situ* hybridization (GISH and FISH) and analyzed by DArTseq genotyping in order to select potentially agronomically useful genotypes for prebreeding purposes. Of the 329,267 high-quality short sequence reads generated, 27,822 SilicoDArT and 8,842 SNP markers specific to *S. cereanum* 1R–7R chromosomes were identified. Heatmaps of the marker densities along the ‘Lo7’ rye reference pseudomolecules revealed subtle differences between the FISH- and DArTseq-based results. This study demonstrates that the “exotic” rye chromatin of *S. cereanum* introgressed into wheat can be reliably identified by high-throughput DArTseq genotyping. The Mv9kr1-’Kriszta’ addition and translocation lines presented here may serve as valuable prebreeding genetic materials for the development of stress-tolerant or disease-resistant wheat varieties.

## Introduction

Wheat (*Triticum aestivum* L.) is one of the most important cereals for human nutrition worldwide. Its grain is used to make bread, pasta, and various bakery products, while its straw is suitable for animal feed and the production of second-generation biofuel (Glithero et al., 2013). By 2050, crop yields will need to be significantly increased due to the world’s rapidly growing population (Ray et al., 2013) and climate change. However, the genetic variability of wheat germplasms has narrowed over thousands of years of cultivation and modern agricultural practices (Jiang et al., 1994; Roussel et al., 2005; van de Wouw et al., 2010), and finding optimal allele combinations within the pangenome of wheat for producing high-yielding cultivars with good nutritional quality and improved heat and drought tolerance, as well as resistance to new pathogen races, remains a major challenge (Moore, 2015).

Hybridizations between bread wheat and related species from primary, secondary, or tertiary gene pools provide a way to increase wheat genetic diversity through chromosome-mediated transfer of wild alleles not existing in cultivated wheat (Jauhar and Chibbar, 1999; Molnár-Láng et al., 2015).

*Secale strictum* (C. Presl) C. Presl (syn. *S. montanum* Guss.) (2*n* = 2*x* = 14, RR) is a wild perennial relative of cultivated rye (*S. cereale* ssp. *cereale*) possessing remarkable genetic diversity (Schreiber et al., 2019), high tillering ability even under suboptimal nutrient availability or water supply, and tolerance to frost (Wang, 1987), drought (Oram, 1996), and high soil aluminum and manganese content (Culvenor et al., 1986). This species is also resistant to rust diseases (Lukaszewski et al., 2001; Schneider et al., 2016) and has a high grain protein content (Kubiczek et al., 1981). Although *S. strictum* is not suitable for cultivation as a forage crop due to its small, scant leaves and “brittle ear” characteristics (Akgün and Tosun, 2004), its perennial habit has been useful in projects aimed at breeding perennial rye varieties with low demands for cultivation and combining the favorable traits of cultivated and wild rye (Reimann-Philipp, 1986).

To facilitate the use of wild genetic variation in breeding programs, two crosses between *S. strictum* and cultivated rye accessions were performed to produce *S. cereanum* varieties: a tetraploid variety (Permontra) in Germany (Reimann-Philipp, 1986) and a diploid variety (Black Mountain) in Australia (Oram, 1996). ‘Kriszta’, ‘Perenne’, and ‘Gergő’ are Hungarian *S. cereanum* cultivars descended from the late 1950s interspecific hybrid *S. cereale* cv. Várda × *S. strictum* ssp. *anatolicum* (Kotvics, 1970). ‘Kriszta’ is cold- and drought-tolerant and resistant to fungal diseases (rusts and powdery mildew) and has a high dietary fiber content (Schneider et al., 2016; Szakács et al., 2020). In order to transfer these traits into hexaploid wheat, cv. Kriszta was involved in a Hungarian introgression breeding program. The cross between the winter wheat line Martonvásári 9 kr1 (Mv9kr1) carrying the recessive crossability allele *kr1* from the spring wheat variety ‘Chinese Spring’ (Molnár-Láng et al., 1996) and ‘Kriszta’ resulted in numerous backcrossed and selfed offspring. Among them, three addition lines disomic for chromosomes 1R, 4R, and 6R, respectively, and the T1BL.1RS translocation line ‘179’ have been identified and phenotyped for grain quality and resistance to foliar diseases (Schneider et al., 2016; Szakács et al., 2020).

The conventional method for visualizing alien chromatin in a host genome is genomic *in-situ* hybridization (GISH), in which labeled DNA probe libraries made from the sheared genomic DNA of multiple known genotypes are competitively hybridized to mitotic chromosomes and visualized (Le et al., 1989; Schwarzacher et al., 1989). GISH can therefore provide information on the number of introgressed chromosomes, the presence of interspecific translocations, and the relative length of the alien chromosome segments. Its combination with fluorescence *in-situ* hybridization (FISH), which relies upon observing chromosomal hybridization profiles of probes designed against specific DNA repeats (Rayburn and Gill, 1985), enables the identification of specific alien chromosomes or chromosome segments in the wheat genetic background (Schneider et al., 2016). In *S. cereale*, a combination of the DNA probe pSc119.2—a member of a 120-bp repeat family (Bedbrook et al., 1980; McIntyre et al., 1990)—and the microsatellite sequence (AAC)_5_ (Cuadrado et al., 2000) is suitable for identifying the entire chromosome complement (Szakács and Molnár-Láng, 2008). However, the high level of polymorphism in the FISH hybridization pattern significantly hampers the identification of the chromosomes of outcrossing rye ‘Kriszta’ carrying both *S. cereale* and *S. strictum* chromatin (Szőke-Pázsi et al., 2022).

The *in-situ* hybridization technique, however, has two significant drawbacks: first, because of its low sensitivity and spatial resolution (deJong et al., 1999), cryptic or micro-introgressions remain undetectable; second, it is laborious and time-consuming, limiting the extent of screening of large wheat-alien prebreeding populations for introgressions.

There are currently several high-throughput and cost-effective molecular techniques available to mitigate these critical limitations. The efficacy of single nucleotide polymorphism (SNP) markers in detecting *Aegilops geniculata* 5M^g^ chromatin introgressed into hexaploid wheat has been reported by Tiwari et al. (2015). The 820k exome-based Axiom SNP array (and its 35k subset) (Winfield et al., 2016; King et al., 2017) has been successfully used to detect introgressions from *Amblyopyrum muticum* (*Aegilops mutica*; King et al., 2017, 2019), *T. monococcum* (Cseh et al., 2019a), *Th. intermedium* (Cseh et al., 2019b), and *T. timopheevii* (Devi et al., 2019). A general disadvantage of fixed SNP arrays is their reliance on known DNA sequence information, and as a result, their use in exotic species is limited. Moreover, array platforms suffer from poor performance in the detection of rare alleles (Ganal et al., 2012; Negro et al., 2019).

DArTseq technology (Sansaloni et al., 2011) is a publicly available, sequence-independent, high-throughput genotyping-by-sequencing (GBS) platform that combines genome complexity reduction steps developed for the microarray hybridization-based DArT method (Jaccoud et al., 2001; Kilian et al., 2005) with next-generation sequencing (NGS) (Elshire et al., 2011). Since the highly methylated repetitive fraction of the DNA samples is eliminated by a methylation-sensitive restriction endonuclease used in the complexity reduction step of the protocol, the majority of high-quality molecular markers generated are low-copy sequences (gene-rich regions). DArTseq markers manifest as short sequences, which are clustered and classified as either dominant SilicoDArT markers (identical sequences that are either present or absent across genotypes) or co-dominant SNP-based markers (homologous sequences containing homo- or heterozygous SNP variants), allowing simultaneous typing of several thousand polymorphic loci spread over a genome in a single experiment. In the case of the genus *Secale*, the DArTseq platform has been successfully used for extending the high-density map of rye and mapping the *Rfc1* male fertility restorer gene on the 4RL chromosome arm (Milczarski et al., 2016); identification of SNPs associated with α-amylase activity, preharvest sprouting, and leaf rust resistance (Rakoczy-Trojanowska et al., 2017); and localization of a gene encoding the transcription factor GAMYB on the 3R chromosome of rye (Bienias et al., 2020) and a new *dw9* dwarfing gene in the 6RL arm (Grądzielewska et al., 2020), alongside the evaluation of genetic diversity in rye inbred lines (Targońska-Karasek et al., 2017) and taxonomic relationships within this genus (Al-Beyroutiová et al., 2016; Targońska-Karasek et al., 2018). Unlike SNP arrays, markers generated by GBS technology, and thus the DArTseq platform, may only be partially reproducible across two genotyping experiments. This issue may become more serious when DArTseq is used to screen different generations of wheat–rye BC populations. The recently published high-quality chromosome-scale genome assembly for rye ‘Lo7’ (Rabanus-Wallace et al., 2021) can overcome these problems by allowing the alignment of DArTseq markers on rye pseudomolecules.

Motivated by the need for a high-throughput and precise screening for the presence of “exotic” rye chromatin in wheat–*S. cereanum* prebreeding populations, the present study used the DArTseq platform in combination with molecular cytogenetic approaches to identify new introgressions. We demonstrated that the SilicoDArT and SNP markers assigned to rye chromosomes using the ‘Lo7’ pseudomolecules, combined with *in-situ* hybridization with DNA repeat and rye genomic probes, are suitable for the precise characterization of *S. cereale* and *S. strictum* chromatin in a wheat-hybrid BC_2_F_8_ generation.

## Materials and methods

### Plant material

Plant genotypes analyzed in this study originate from the BC_2_F_8_ generation of the cross between the wheat (*Triticum aestivum* L.; BBAADD, 2*n* = 6*x* = 42) line Mv9kr1 and the perennial rye (*Secale cereanum*; RR, 2*n* = 2*x* = 14) cultivar Kriszta carried out in 2001. The development of this population is described in detail in Schneider et al. (2016) and Szakács et al. (2020). Additional plant materials also involved in the DArTseq marker analysis include Mv9kr1 and ‘Kriszta’ as parental genotypes of the BC_2_F_8_ generation; *S. cereale* L. (RR, 2*n* = 2*x* = 14) cv. Várda and *S. strictum* (C. Presl) C. Presl. ssp. *anatolicum* (Boiss.) K. Hammer (RR, 2*n* = 2*x* = 14) as parental genotypes of ‘Kriszta’; *T. aestivum* L. cv. Chinese Spring (CS) and *S. cereale* L. cv. Imperial as parental genotypes of the CS-’Imperial’ additions; CS-’Imperial’ (wheat–rye) disomic addition series (1R–7R) (Driscoll and Sears, 1971) and CS-’Imperial’ ditelosomic addition lines 1RS, 1RL, 2RL, 3RS, 4RS, 4RL, 5RL, 6RL, and 7RS from the Wheat Genetics Resource Center (Kansas State University, USA); *S. cereale* L. cv. Petkus; Martonvásár T1BL.1RS wheat cultivars Mv Magdaléna and Mv Matador carrying 1RS of ‘Petkus’ rye origin; and *S. cereale* L. cv. Lovászpatonai and Mv9kr1-’Lovászpatonai’ disomic 4R addition line (Szakács et al., 2016).

The BC_2_F_8_ plants were grown and their spike morphology was documented in the field nursery “Tükrös” (Martonvásár, Hungary; 47°18′40″N, 18°46′56″E) in 2022.

### DArTseq marker development and analysis

Genomic DNA samples were extracted from plants grown in Jiffy peat pots at the four-leaf stage using the QuickGene Mini80 extraction system and QuickGene DNA tissue kit S (FujiFilm, Japan). Thirty-microliter aliquots of the DNA samples (70 ng/μl each) were sent to Diversity Arrays Technology Pty, Ltd. (University of Canberra, Australia) for genotyping. Rye DArTseq version 1.0. was used to generate high-quality, 69-bp sequence reads with a call rate >95%. To discard wheat-specific DArTseq markers and identify rye-specific ones, data filtering was performed. For this, the dominant SilicoDArT markers were scored as “1” or “0” reflecting the presence or absence of a marker in the genomic representation of each sample, while the co-dominant SNP markers were scored as “−,” “0,” “1,” and “2” representing the absence of a marker, reference allele (Rye_v2; provided by Diversity Arrays Technology Pty Ltd.) only, alternative allele, and both reference and alternative alleles, respectively. In the case of the SNPs, markers having common match sequences at the start position were used to create consensus markers by combining their sequences. Pairwise alignment of the sequences was carried out by using the MEGA6 (Molecular Evolutionary Genetics Analysis v. 6.0) software, and then the consensus sequences were generated on the Geneious v. R11.1.5 bioinformatics software platform.

Genotype data of the samples containing rye chromatin were then analyzed to select putative 1R–7R-specific markers. The chromosome scale ‘Lo7’ reference sequence assembly (Rye_Lo7_2018_v1p1p1) of *S. cereale* (Rabanus-Wallace et al., 2021) was used to identify the chromosomal location of these markers. Trimmed sequences of the putative 1R–7R markers were used as queries for homology searches performed using the *blastn* package of the BLAST Command Line Application 2.9.0 (https://blast.ncbi.nlm.nih.gov/) with the following parameters: E-value = 1e−5; -max_target_seqs = 1; -max_hsps = 1. The distribution of SilicoDArT and SNP markers in the non-coding and coding regions of the Lo7 rye reference genome was determined using various BLASTn identity threshold (IDT) parameters (70%, 85%, 93%, and 95%) and an alignment length (AL) of ≥67 nucleotides. Marker positions were compared to the positions of Lo7 gene models (Rye_Lo7_2018_v1p1p1.59.gff3.gz; Ensemble Plants, Release 59) using GffCompare of GFF utilities (Pertea and Pertea, 2020). The alignment of the coding region-specific marker sequences to the reference transcripts was investigated using the classification of GffCompare as “contained in reference (intron compatible)” (c), “single exon transfrag partially covering an intron, possible pre-mRNA fragment” (e), “other same strand overlap with reference exons” (o), “exonic overlap on the opposite strand (like o or e but on the opposite strand)” (x), “fully contained within a reference intron” (i), “possible polymerase run-on (no actual overlap)” (p), and “none of the above (unknown, intergenic)” (u).

The density of the 1R–7R-specific marker positions across the pseudomolecules was visualized as heatmaps using the MeV (Multiple Experiment Viewer) 4.9.0. software.

### Preparation of metaphase chromosome spreads

The seeds of the Mv9kr1-’Kriszta’ BC_2_F_8_ progeny plants were germinated on wet filter paper in Petri dishes at room temperature for 24 h and incubated at 4°C for 48 h and then at 25°C for 26 h. Excised root tips were kept in ice water at 4°C for 24 h and subsequently in Carnoy’s I fixative (1 part glacial acetic acid and 3 parts absolute ethanol) at 37°C for 1 week, stained in 1% acetocarmine solution for 2 h, and then fixed again. Root tips were squashed in 45% acetic acid. After removing the coverslips, the preparations were dehydrated in a graded series (70% to 100%) of ethanol, air-dried overnight, and stored at −20°C until use.

### Fluorescence *in-situ* hybridization and genomic *in-situ* hybridization

The highly repetitive rye DNA sequence pSc119.2 was amplified from the genomic DNA of *S. cereale* using the PCR protocol described by Contento et al. (2005) and labeled with biotin-14-dATP (Invitrogen, Carlsbad, USA) by nick translation following the manufacturer’s instructions. The total genomic DNA of ‘Kriszta’ rye was labeled with digoxigenin-11-dUTP-t (Roche, Mannheim, Germany) and used as a probe for GISH. FISH and GISH were performed simultaneously. The hybridization mixture (30 μl per slide) contained 30 ng of labeled rye genomic DNA, labeled DNA probes (40 ng each), 50% v/v formamide, 2× SSC (0.15 mol/L of NaCl plus 0.015 mol/L of sodium citrate), 10% w/v dextran sulfate, 0.1% w/v sodium dodecyl sulfate, and 1.4 µg of salmon sperm DNA. Chromosome preparations together with the hybridization mix were denatured on a hot PCR plate at 80°C for 2 min and hybridized at 37°C overnight in a humid slide incubator. The biotin and digoxigenin signals were detected with streptavidin-Alexa Fluor 488 (Thermo Fischer Scientific, Waltham, MA, USA) and anti-digoxigenin-rhodamine (Roche Diagnostics, Mannheim, Germany), respectively. The chromosome preparations were counterstained with 1 µg/ml of 4′,6-diamidino-2-phenylindole (DAPI; Amersham, Germany) and examined with a Zeiss Axio Imager 2 microscope (Carl Zeiss Microscopy GmbH, Jena, Germany). Images were acquired with a Zeiss AxioCam MRm CCD camera using AxioVision SE64 Rel. 4.9.1 software (Carl Zeiss Microimaging GmbH, Jena, Germany).

## Results

### Assignment of DArTseq markers to rye chromosomes

DArTseq genotyping of the BC_2_F_8_ generation of the cross between Mv9kr1 and ‘Kriszta’ resulted in a total of 329,267 high-quality reads. For sequence similarity searches, the trimmed (69 bp) sequences of 258,090 SilicoDArT markers and 71,177 SNP markers were blasted against chromosome-specific rye pseudomolecules. During the analysis, we discovered that the genetic diversity between *S. strictum* and the domesticated *S. cereale* ‘Lo7’ is relatively high; as a result, we used an IDT of 70% to map markers to the rye pseudomolecules and screen the BC_2_F_8_ generation for the presence of rye chromatin. Markers having an alignment with less than 70% identity score and those specific for several loci were discarded. Further filtering of the obtained 35,694 SilicoDArT and 27,321 SNP markers was carried out differently.

To select rye-specific sequences for visualization of introgressed rye chromatin in wheat background, SilicoDArT markers with alleles also present (scored as “1”) in wheat (Mv9kr1 and CS) were excluded from the study. From the selected 31,684 markers, those absent from both the cultivated and wild ryes (1R–7R scored as “0”) were also discarded. Out of the 30,702 markers obtained, after retaining only one marker from those that had the same start position during the sequence alignment, 27,822 markers remained (**Table 1**; **Supplementary Data Sheet 1**).

**Table 1:** Distribution among the *Secale cereale* ‘Lo7’ pseudomolecules of the *Secale cereanum* rye-specific SilicoDArT and SNP markers.

Marker type	Chromosome							Total
	1R	2R	3R	4R	5R	6R	7R	
SilicoDArT	3,381	4,373	3,512	4,359	4,103	4,467	3,627	27,822
SNP	1,208	1,504	1,241	1,159	1,417	1,074	1,239	8,842

In the case of the SNPs, after discarding those having at least one wheat (Mv9kr1 or CS) allele identical to one of the rye (cvs. Imperial, Petkus, Lovászpatonai, Várda, *S. strictum* ssp. *anatolicum*, or cv. Kriszta) alleles (taking into consideration that an allele scored as “2” comprises alleles both “0” and “1”) and those absent from the rye genotypes (scored as “–”), out of the 71,177 markers, 14,042 rye-specific markers were selected. There were 5,208 SNPs that were mapped to 5,208 unique positions on the ‘Lo7’ pseudomolecules, while 8,834 SNPs were mapped to 3,634 positions. These 8,834 redundant markers formed 3,634 groups of two to seven markers with identical trimmed sequences and, as a result, identical positions on the ‘Lo7’ chromosome. In order to eliminate the redundant markers, we chose one marker from each group as the “delegated marker” to represent the genomic positions of the redundant marker groups (**Supplementary Data Sheet 2**). Finally, the 5,208 individual SNPs and the 3,634 delegated SNPs (a total of 8,842 SNPs; **Table 1**) were used to screen the plant material.

To test the reliability of the marker analysis at a higher stringency, we also ran the analysis with a match length of ≥67 nucleotides and IDTs of 85%, 93%, and 98%. **Table 2** summarizes the results showing the number of SilicoDArT markers, which decreased from 27,822 by approximately half, reaching 14,270 and 14,007 at IDTs 70%–93% and 98%, respectively. The 8,842 SNP markers lowered to 5,048 at IDTs 70% and 85%, 4,874 at 93%, and 2,708 at 98% stringency cutoff.

**Table 2:** Distribution of SilicoDArT and SNP markers filtered between the non-coding and coding regions of the Lo7 rye reference genome (Rye_Lo7_2018_v1p1p1) using various threshold parameters [ID and alignment length (AL)].

	ID (%) + AL (nucleotides)				
	70% + Ø	70% + ≥67	85% + ≥67	93% + ≥67	98% + ≥67
SilicoDArT marker sequences					
Non-coding (p*+u*)	19,660 (70.6%)	9,843 (68.9%)	9,843 (68.9%)	9,843 (68.9%)	9,648 (68.8%)
**Coding (total)**	8,162 (29.4%)	4,427 (31.1%)	4,427 (31.1%)	4,427 (31.1%)	4,359 (31.2%)
c*	2,438 (29.8%)	1,166 (26.3%)	1,166 (26.3%)	1,166 (26.3%)	1,151 (26.4%)
e*	248 (3.2%)	182 (4.1%)	182 (4.1%)	182 (4.1%)	178 (4.0%)
o*	893 (10.9%)	513 (11.5%)	513 (11.5%)	513 (11.5%)	502 (11.5%)
x*	3,227 (39.5%)	1,716 (38.7%)	1,716 (38.7%)	1,716 (38.7%)	1,690 (38.7%)
i*	1,356 (16.6%)	850 (19.2%)	850 (19.2%)	850 (19.2%)	838 (19.2%)
**Total**	27,822 (100%)	14,270 (100%)	14,270 (100%)	14,270 (100%)	14,007 (100%)
SNP marker sequences					
Non-coding (p*+u*)	2,507 (28.4%)	1,223 (24.3%)	1,223 (24.3%)	1,165 (24.0%)	568 (21.0%)
**Coding (total)**	6,335 (71.6%)	3,825 (75.7%)	3,825 (75.7%)	3,709 (76.0%)	2,140 (79.0%)
c*	2,461 (38.8%)	1,472 (38.4%)	1,472 (38.4%)	1,429 (38.5%)	833 (38.9%)
e*	75 (1.1%)	53 (1.3%)	53 (1.3%)	52 (1.4%)	31 (1.4%)
o*	597 (9.4%)	370 (9.6%)	370 (9.6%)	361 (9.7%)	200 (9.3%)
x*	2,990 (47.1%)	1,795 (46.9%)	1,795 (46.9%)	1,738 (46.8%)	1,003 (46.8%)
i*	212 (3.3%)	135 (3.5%)	135 (3.5%)	129 (3.4%)	73 (3.4%)
**Total**	8,842 (100%)	5,048 (100%)	5,048 (100%)	4,874 (100%)	2,708 (100%)

The relationships between the coding region-specific markers and the reference transcripts were obtained and classified* by GffCompare.

*c: contained in reference (intron-compatible).

*e: single exon transfrag partially covering an intron, possible pre-mRNA fragment.

*o: other same strand overlap with reference exons.

*x: exonic overlap on the opposite strand (like o or e but on the opposite strand).

*i: fully contained within a reference intron.

*p: possible polymerase run-on (no actual overlap).

*u: none of the above (unknown, intergenic).

Interestingly, we found that the ratio of gene-coding to non-coding sequences was 29.4%:70.6%, and it remained largely constant at higher stringency conditions in the case of SilicoDArT markers. In contrast, SNP markers were located more frequently on coding regions, as the ratio of coding to non-coding sequences was 70.6%:29.4% and it was even higher at 98% IDT (79%:21%).

For both the SilicoDArT and the SNP markers specific for coding regions, the most common alignment to the reference gene model was type “x” (“exonic overlap on the opposite strand”), followed by “c” (i.e., intron-compatible matches) (**Table 2**). The third most common matching type for SilicoDArT markers was “i” (i.e., fully aligned in a reference intron), followed by “o,” while the order was reversed for SNP markers. For both the SilicoDArT and the SNP markers, the transcript classification code “e” was the least common. The percentage distribution of the transcript classification codes did not significantly change with increasing stringency levels for either SilicoDArT or SNP markers (**Table 2**).

### Molecular and cytogenetic screening for the presence of “exotic” rye chromatin

In order to visualize the introgressed rye chromatin in the wheat BC_2_F_8_ generation, the distribution of rye-specific SilicoDArT or SNP markers on the rye chromosomes was exhibited on a heatmap.

However, the number of markers filtered at stringent 93% IDT condition (**Table 2**; **Supplementary Data Sheets 3**-**4**) and the low marker density hampered the reliable detection of rye chromatin (**Supplementary Figures 1**, **2**). Especially in the case of 5RS and 6RS, the marker density was so low that the centromeric region was indistinguishable from the rest of the short chromosome arms.

**Figure 1:**
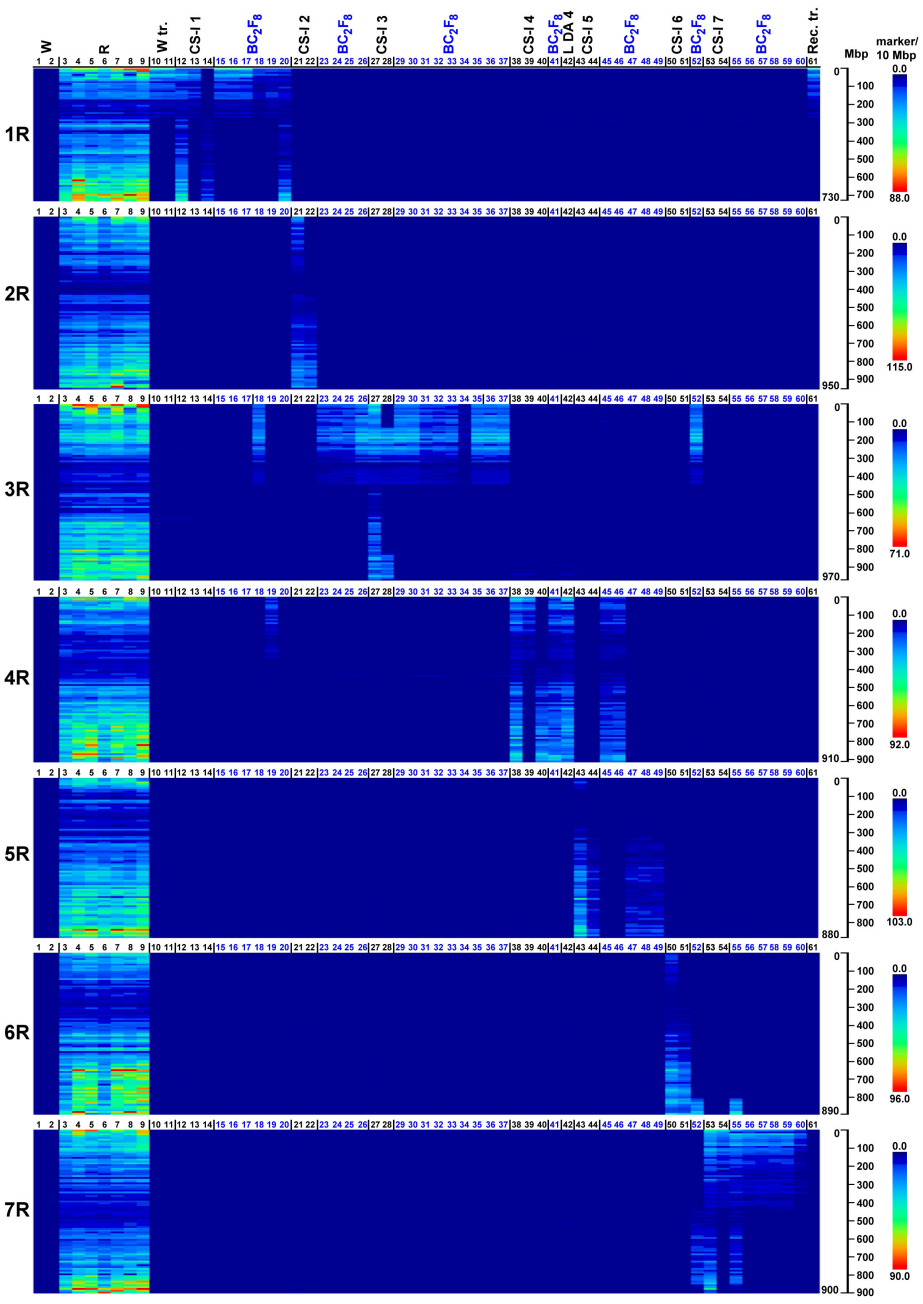
Heatmap representation of SilicoDArT marker densities obtained on wheat genotypes with (W tr.) or without (W) 1BL.1RS translocation, wild and domesticated rye genotypes (S), reference wheat–rye disomic and ditelosomic chromosome additions (CS-I 1–7, L DA 4), and Mv9kr1-Kriszta BC_2_F_8_ (BC_2_F_8_) lines on each pseudomolecule of rye (1R–7R). Reads from the investigated genotypes (detailed in **Table 3**) were aligned to the ‘Lo7’ chromosome-scale assembly at IDT 70% and ordered along the chromosomes by the start position. The numbered scales show chromosome lengths in megabase pairs (Mbp), and the colored scales depict marker densities (number of markers per 10 Mbp).

**Figure 2:**
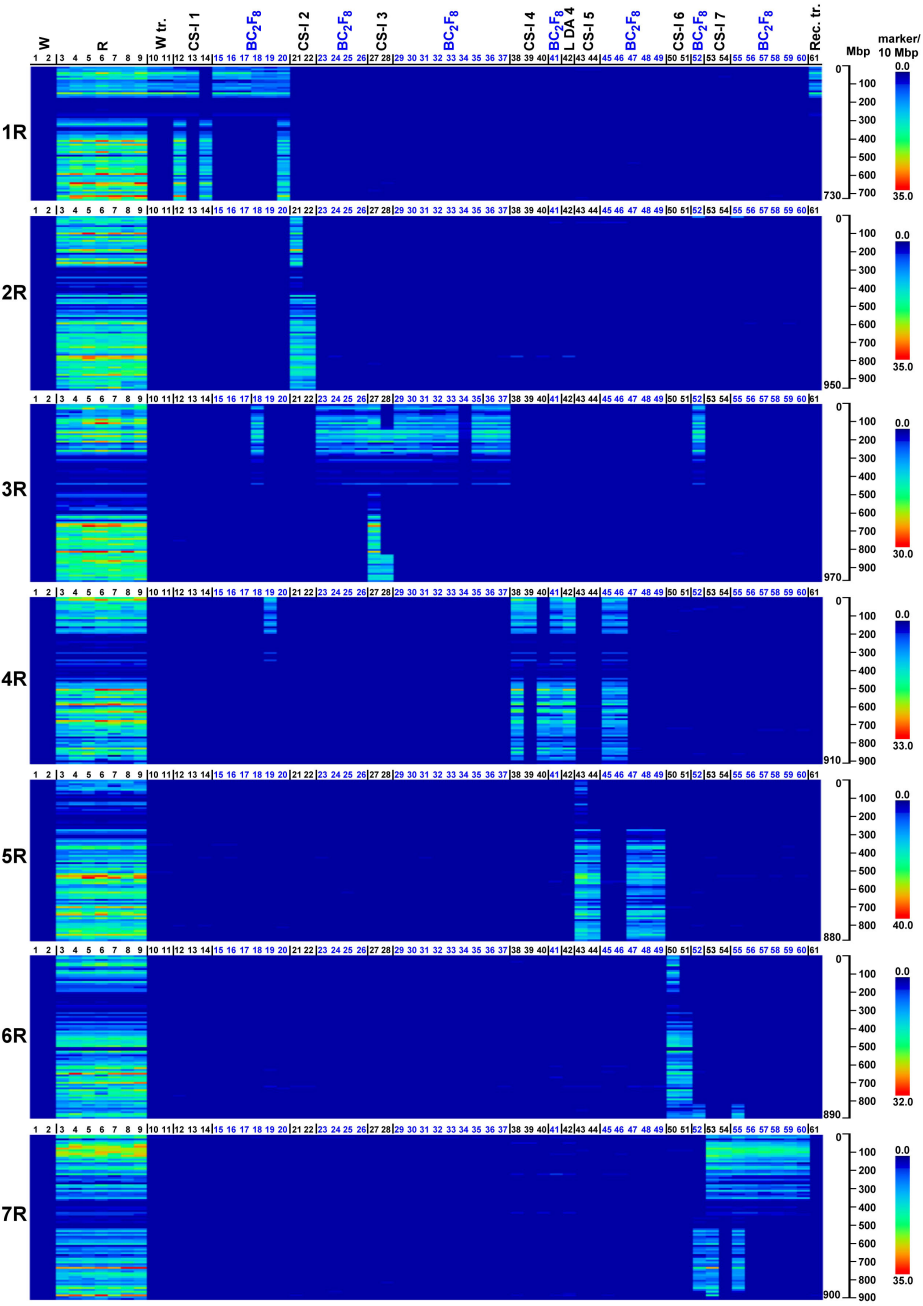
Heatmap representation of SNP marker densities obtained on wheat genotypes with (W tr.) or without (W) 1BL.1RS translocation, wild and domesticated rye genotypes (S), reference wheat–rye disomic and ditelosomic chromosome additions (CS-I 1–7, L DA 4), and Mv9kr1-Kriszta BC_2_F_8_ (BC_2_F_8_) lines on each pseudomolecule of rye (1R–7R). Reads from the investigated genotypes (detailed in **Table 3**) were aligned to the ‘Lo7’ chromosome-scale assembly at IDT 70% and ordered along the chromosomes by the start position. The numbered scales show chromosome lengths in megabase pairs (Mbp), and the colored scales depict marker densities (number of markers per 10 Mbp).

At the 70% stringency threshold, the distributions of the 27,822 SilicoDArT and 8,842 SNP markers across rye chromosomes 1R–7R are generally highly concordant and clearly detect the rye chromatin introgressed in the wheat. As expected, no rye-dominant SilicoDArT markers or rye-associated SNPs (we refer to both collectively as rye markers henceforth) were observed in either wheat genotypes (Mv9kr1 and CS, corresponding to lanes 1 and 2 in **Figures 1**, **2**). (The code numbers and the abbreviations in the figures are explained in **Table 3**). Conversely, rye accessions (e.g., lanes 3 to 9) are generally rich in rye marker across the entire chromosome with the exception of pericentromeres. This pericentromeric tendency is confirmed by the rye marker binding profiles of ditelosomic samples, e.g., that of 1RS (lane 13) and 1RL (lane 14). SilicoDArT markers are more abundant at the terminal positions, with the exception of chromosome arms 4RL and 6RL in which the marker density is also high interstitially, and 5RS and 6RS, where the marker frequency is low. SNP markers, however, are widely distributed on all chromosomes, both interstitially and terminally, except for arms 5RS and 6RS (as is the case with the SilicoDArT markers).

**Table 3:** Code numbers and abbreviations of the genotypes analyzed by fluorescence *in-situ* hybridization (FISH) and DArTseq markers.

Code No.	Abbreviation	Genotypes		
In the heatmaps			FISH	DArTseq analysis
1	W	*T. aestivum* ‘Mv9kr1’	**–**	**–**
2		*T. aestivum: CS*		
3	S	*S. strictum* ssp. *anatolicum*	**–**	1R-7R
4–5		*S. cereale* ‘Várda’		
6		*S. cereanum* ‘Kriszta’		
7		*S. cereale* ‘Imperial’		
8		*S. cereale* ‘Petkus’		
9		*S. cereale* ‘Lovászpatonai’		
10	W tr.	T1BL.1RS wheat ‘Mv Magdaléna’	T1BL.1RS	1RS
11		T1BL.1RS wheat ‘Mv Matador’		
12	CS-I 1	CS-‘Imperial’	DA 1R	1R
13			DT 1RS	1RS
14			DT 1RL	1RL
15	BC_2_F_8_	Mv9kr1-‘Kriszta’	T1BL.1RS a	1RS
16			T1BL.1RS b	
17			T1BL.1RS c	
**18**			**DA 1R**	**1RS.3RS**
**19**				**1RS.4RS**
20				1R
21	CS-I 2	CS-‘Imperial’	DA 2R	2R
22			DT 2RL	2RL
**23–25**	BC_2_F_8_	Mv9kr1-‘Kriszta’	**Twheat.2RL(2RS)**	**3RS**
**26**			**MA 2RS iso**	
27	CS-I 3	CS-‘Imperial’	DA 3R	3R
**28**			**DT 3RS**	**3RS^del^.3RL^del^**
29, 30	BC_2_F_8_	Mv9kr1-‘Kriszta’	MA 3RS iso	3RS
**31–35**			**Twheat.3RL**	3RS*
**36, 37**			**DA 3R**	**3RS**
38	CS-I 4	CS-‘Imperial’	DA 4R	4R
39			DA 4RS	4RS
40			DA 4RL	4RL
41	BC_2_F_8_	Mv9kr1-‘Kriszta’	DA 4R	4R
42	L DA 4	Mv9kr1-‘Lovászpatonai’	DA 4R	4R
43	CS-I 5	CS-‘Imperial’	DA 5R	5R
44			DT 5RL	5RL
**45, 46**	BC_2_F_8_	Mv9kr1-‘Kriszta’	**DA 5R**	**4R**
47–49			T1BL.5RL	5RL
50	CS-I 6	CS-‘Imperial’	DA 6R	6R
51			DT 6RL	6RL
**52**	BC_2_F_8_	Mv9kr1-‘Kriszta’	**DA 6R**	**3RS.7RL-6RL**
53	CS-I 7	CS-‘Imperial’	DA 7R	7R
54			DT 7RS	7RS
**55**	BC_2_F_8_	Mv9kr1-‘Kriszta’	**DA 7R**	**7RS.7RL-6RL**
**56**			DT 7RS**(7RL)**	**7RS**
**57–60**			T4BL.7RS(**7RL)**	
61	Rec. tr.	‘Mv Magdaléna’/Mv9kr1/‘Kriszta’	Recombined T1BL.1RS	1RS

a, b, and c: lines 179, C5, and D5, respectively, described in Szakács et al. (2020); *: except for lane 34 (no markers); in bold: recognized as different by FISH and DArTseq analysis.

DA, disomic addition; DT, ditelosomic addition; MA, monosomic addition; T, translocation; iso, isochromosome.

**Figure 3:**
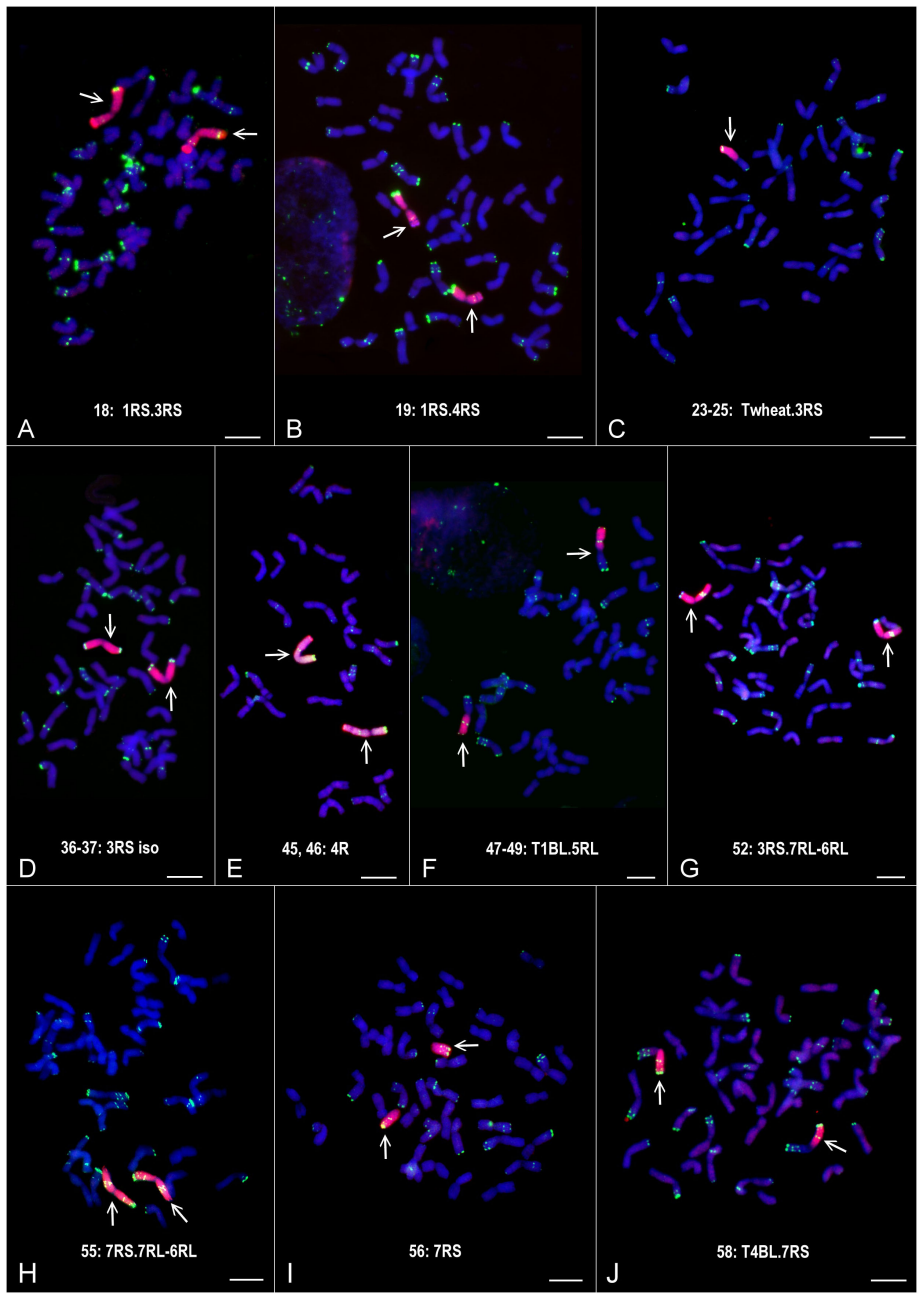
Partial or complete chromosome sets of the Mv9kr1-‘Kriszta’ disomic addition **(A, B, D, E, G)** and **(H)**, ditelosomic addition **(I)**, and translocation **(C, F)** and **(J)** lines identified by DArTseq markers. The GISH (rye total genomic DNA) and FISH (pSc119.2) signals are red and green, respectively. Bars: 10 µm.

**Figure 4:**
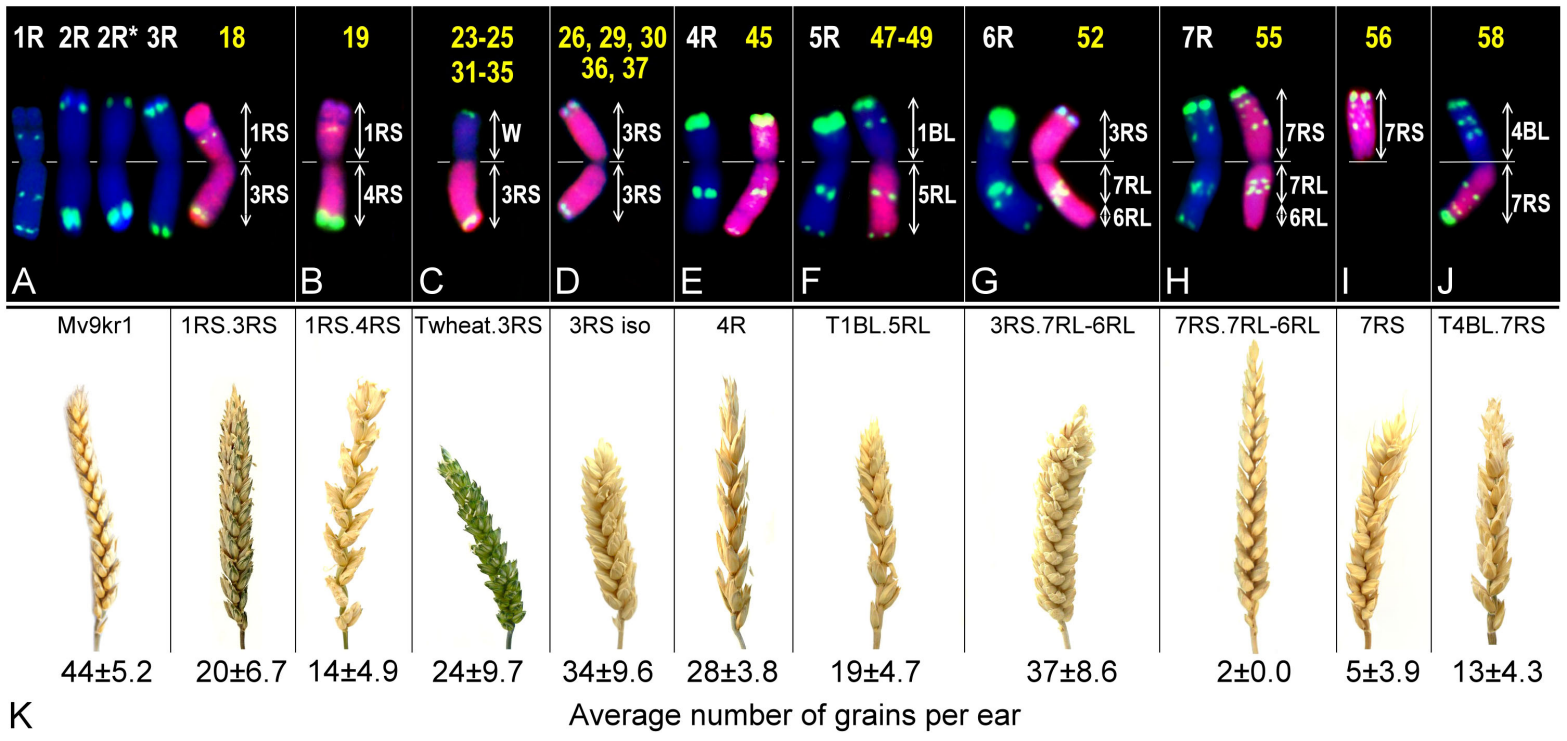
Composition and localization (lane number) on the heatmaps **(A–J)** and ear morphology **(K)** of the parental wheat line Mv9kr1 and the Mv9kr1-‘Kriszta’ lines shown in **Figure 3**. The ears are represented in proportion to their actual size. The trait grain number per ear was calculated for 10 ears of each BC_2_F_8_ genotype. White letters in the upper row indicate ‘Kriszta’ chromosomes with the characteristic pSc119.2 FISH pattern. *: polymorphic pattern.

In general, the marker analysis confirmed the genetic constitution of the plants from which the DNA samples were isolated; however, minor discrepancies between the results obtained by FISH and the marker analysis are occasionally observed. The Mv9kr1-’Kriszta’ BC_2_F_8_ progeny plant (lane 18) was identified by the presence of a short chromosome arm with satellite and by FISH [considering the high pSc119.2 signal polymorphism of ‘Kriszta’ 1R (Szakács et al., 2020)] as a putative 1R disomic addition line. The marker analysis shows that the long arm of the introgressed rye chromosome is 3RS (**Figures 1**, **2**, **3A**, **4A**). The chromosomes in the disomic addition in lane 19 were also supposed to be 1R, but according to the DArTseq markers, they are T1RS.4RS translocations (**Figures 1**, **2**, **3B**, **4B**). The identification of the 2RS, 2RL, 3RS, and 3RL chromosome arms of ‘Kriszta’ owing to their similar pSc119.2 patterns is difficult (**Figure 4A**), particularly when they participate in translocations.

Based on the position of the FISH signal and the size of the translocated chromosome arm, genotypes analyzed in lanes 23–25 were presumed to be Twheat.2RL (or Twheat.2RS), while those presented in lanes 31–35 were described as Twheat.3RL monosomic translocations. Similarly, the plant in lane 26 was presumed to be a genotype carrying a 2RS isochromosome. The genebank-originated accession presented in lane 28 was believed to be ditelosomic 3RS, but according to the present study, the telocentric chromosome pair is a composition of a proximal segment (including the centromere) of 3RS (3RS^del^) and a distal segment (including the telomeric region) of 3RL (3RL^del^). The BC_2_F_8_ progeny plants in lanes 36 and 37 were identified as putative disomic 3R additions. The DArTseq markers reveal that all the abovementioned genotypes contain 3RS arms (**Figures 3C, D**, **4C, D**). The chromosomes of plants in lanes 45 and 46 were found to be 5R, based upon their morphology (arm ratio), as well as a strong interstitial and a faint terminal pSc119.2 signal on the long arm. DArTseq markers argue they are unambiguously 4R chromosomes showing the same SNP marker densities as the Mv9kr1-’Kriszta’ disomic 4R addition line in lane 41. These density patterns differ from the CS-’Imperial’ (lane 38) and Mv9kr1-’Lovászpatonai’ (lane 42) 4R additions (**Figures 2**, **3E**, **4E**), reflecting differences between the *S. cereale* and *S. cereanum* R genomes. However, the marker analysis reveals that the chromosome arms of the translocations in lanes 47–49 with the same pSc119.2 signals are 5RL (**Figures 1**, **2**, **3F**, **4F**). Chromosomes of the disomic addition line (lane 52), which were presumed to be 6R based on FISH, were identified by the markers as translocations carrying a 3R short arm and a long arm composed of a longer 7RL and, terminally, a shorter 6RL chromosome segment (**Figures 1**, **2**, **3G**, **4G**). The same rearranged long arm was detected in a disomic addition line (lane 55) identified by FISH as 7R (**Figures 1**, **2**, **3H**, **4H**). The marker analysis also revealed that the two telocentric rye chromosomes in the genotype shown in lane 56 (**Figures 1**, **2**, **3I**, **4I**), as well as the rye chromosome segments translocated onto the 4BL wheat chromosome arm in another BC_2_F_8_ progeny plant (lane 58) (**Figures 1**, **2**, **3J**, **4J**), are 7RS arms.

### Ear morphology of the wheat–*Secale cereanum* introgressions

A comparison of the ear morphology of the genotypes described above suggests that some rye chromosome arms have a greater effect on this trait than others (**Figure 4K**). The ears of the plants carrying 3RS arms are more compact than those of the genotypes carrying 4RS, 5RL, 6RL, 7RS, and 7RL. In the case of the disomic addition line 1RS.3RS, the ears are more compact than in the case of 1RS.4RS. The ears of this latter genotype are similar to those of the disomic 4R addition being elongated and loose. Thus, it seems that 4RS has a stronger influence on the ear morphology than 1RS. In terms of the fertility expressed as seed number per ear (**Figure 4K**), the genotypes can be arranged in the following order: 7RS.7RL-6RL disomic addition (with the lowest value) – ditelosomic 7RS – T4BL.7RS – 1RS.4RS disomic addition – T1BL.5RL – 1RS.3RS disomic addition – Twheat.3RS – disomic addition of the 3RS isochromosome – 3RS.7RL-6RL disomic addition.

## Discussion

The development of high-throughput genotyping-by-sequencing has greatly increased the sensitivity of introgression detection in crop plants (Scheben et al., 2017). A great advantage of the DArTseq genome profiling platform over the hybridization-based DArT method (e.g., microarray-based DArT) is its flexibility, as it does not require prior sequence information. The present study further demonstrated the efficiency of DArTseq technology for screening a wheat introgression breeding population for the presence of exotic rye (a hybrid of *S. cereale* and *S. strictum*) chromatin.

By mapping reads generated by high-throughput genotyping systems onto publicly accessible high-quality genome assemblies and chromosome-scale pseudomolecules for *Hordeum vulgare* (Mascher et al., 2017), *Aegilops tauschii* (Luo et al., 2017), and several *Triticum* species (Avni et al., 2017; Appels et al., 2018; Ling et al., 2018; Maccaferri et al., 2019; Zhu et al., 2021), it was possible to identify chromosome rearrangements between and within species.

High-quality chromosome-scale reference genome assemblies for *S. cereale* have also become available recently for the inbred line ‘Lo7’ (Rabanus-Wallace et al., 2021) and another for the elite Chinese cultivar ‘Weining’ (Li et al., 2021). These genomic resources shed light on the evolution of cultivated rye and the mechanisms of genome structure modifications (Rabanus-Wallace et al., 2021), as well as the physical organization of complex loci and gene expression features in the rye (Li et al., 2021). The availability of Lo7 pseudomolecules also allowed us to determine the genomic positions of DArT marker sequences obtained in the parental and control rye genotypes. In the present study, ~30% of the SilicoDArT markers were located in coding regions, while ~70% of the SNP markers were gene-specific. This ratio was higher than those obtained in common bean, where 43.3% of the SNP DArT markers were gene-specific (Valdisser et al., 2017), or the ratio (~60%) obtained in the genus *Psidium* (Grossi et al., 2021). In connection with this, we also found a general trend of increasing DArT marker density from centromeric–pericentromeric regions to telomeres. This phenomenon corresponds to the expectation that marker coverage will be higher in distal regions of the chromosomes with active gene expression (Akhunov et al., 2003). However, Bauer et al. (2017) discovered that rye chromosomes contain numerous genes in their centromeric and pericentromeric regions. Given that the methylation-sensitive *PstI* and frequently cutting *TaqI* restriction endonucleases used for genome complexity reduction generate low-copy (gene-rich) sequences, markers should theoretically be present in these chromosome regions. The low abundance of the markers in the pericentromeric regions can most likely be explained by the small number of low-copy sequences compared to distal regions. An alternative explanation could be the high methylation level of the proximal pericentromeric regions, which are known to be strongly heterochromatic (Kalinka and Achrem, 2020; Schreiber et al., 2022). The high methylation level in these regions can inhibit the methylation-sensitive *PstI* function, resulting in a relative lack of cleavage sites and, consequently, a decrease in marker density.

Differences in marker density between the proximal and distal parts of chromosome arms, on the other hand, did not limit the detection of individual rye chromosomes or the identification of small intra- or interchromosomal rearrangements that could not previously be detected by PCR-based markers or *in-situ* hybridization (Schneider et al., 2016).

Recently, Keilwagen et al. (2019) examined diverse barley and wheat collections to predict the size, frequency, and identity of introgressed chromosome regions, using the bioinformatics approach GBS coverage analysis. From the sequencing coverage profiles, which depict patterns of increased and decreased sequence coverage, the authors identified chromosomal alterations on a genome-wide scale, including introgressions, copy number variations, and modifications in DNA methylation. The same sequence coverage method was also applicable to identify alien chromatin from wild *Triticum* and *Aegilops* species in wheat chromosomes 2A, 2B, 2D, 3D, and 4A and to predict putative donor species (Keilwagen et al., 2022).

In the present study, we used heatmap visualization of the marker density to detect chromatin from the *S. cereanum* chromosomes 1R, 3RS, 4R, 5RL, 6RL, and 7RL introgressed into bread wheat. The resolution of rye-specific DArT markers whose chromosomal position was determined under a less stringent condition (70% identity percent) was sufficient to identify rye chromatin transferred into the Mv9kr1 wheat background. However, some discrepancies were found between the cytogenetic and DArTseq identification of *S. cereanum* chromosomes in some BC_3_F_8_ lines. We believe that the main reason for these discrepancies was not the low cutoff used to determine the genomic position of the markers and, thus, the poor determination of the marker positions. This opinion is supported by the results of 16 Chinese Spring (CS)–Imperial (I) disomic and ditelosomic addition lines (CS-I 1–7) (**Figures 1**, **2**), genotyped together with the BC_3_F_8_ population and representing all of the rye chromosomes (1R–7R) and several chromosome arms (1RS, 1RL, 2RL, 3RS, 4RS, 4RL, 5RL, 6RL, 7RS) (**Table 3**). These reference wheat–rye cytogenetic stocks, similar to many genetic studies aimed to identify molecular markers on rye chromosomes (Qiu et al., 2016; Ma et al., 2020; Li et al., 2022), were used to validate if rye-specific marker positions were well determined on the Lo7 reference sequence. **Figures 1**, **2** demonstrate that the corresponding rye chromosomes or chromosome arms could be detected perfectly in 15 of the 16 reference CS-Imperial cytogenetic stocks. A 3RS-3RL rearranged structure was only identified for the 3RS ditelosomic line, but this is because of the rearranged structure of the chromosome arm in this specific genotype. (If the wrong map position of the markers was the reason, the distal half of 3RS would be missing from all 3RS-carrying genotypes.) We have to note that similar results were obtained with DArTseq markers mapped under a more stringent condition (93% IDT), but the marker coverage of the chromosomes was so low that visualizing the entire length of rye chromosomes and chromosome arms became problematic. If the genomic position of the rye-specific markers was incorrectly identified, the identified rye chromatin in the CS-Imperial lines would differ from the “official” rye chromosome content.

In our opinion, the most likely cause of discrepancies between cytogenetic and DArTseq identification of the rye chromosome in the BC_3_F_8_ lines is cytogenetic misidentification. In the present study, we used the pSc119.2 repeat as a FISH probe to identify rye chromosomes based on their hybridization pattern, which is mainly composed of telomeric and subtelomeric bands. However, our previous study revealed a high level of pSc119.2 hybridization pattern polymorphism on the chromosomes of *S. cereanum* ‘Kriszta’ (Szőke-Pázsi et al., 2022). The inability of GISH to detect intragenomic rearrangements in rye, combined with the existence of similar FISH patterns on different chromosome arms and FISH polymorphism on homologs, leads to an incorrect cytogenetic identification of rye chromosomes. These results highlight one of the advantages of using high-resolution marker systems together with the rye reference assembly; when combined with GISH, they provide a more detailed insight into the structure of transferred rye chromatin and are suitable for detecting intragenomic structural changes of rye chromosomes.

We also speculated on when the intragenomic rye chromosome rearrangements took place. We think that these rearrangements (like 1RS.3RS, 1RS.4RS) formed most likely after the first *S. cereale* × *S. strictum* crosses and/or during the breeding of the ‘Kriszta’ variety. This idea seems to be supported by the polymorphic karyotype of ‘Kriszta’ (Szőke-Pázsi et al., 2022). However, the planned development of a chromosome-scale reference genome sequence for ‘Kriszta’ will provide a more detailed understanding of the genome structure of this exotic *S. cereale* × *S. strictum* hybrid, as well as its genome relationship with domesticated *S. cereale*. Because ‘Kriszta’ is a source of several agronomically important traits (resistance to yellow rust and leaf rust, yield-increasing effect, grain quality) (Schneider et al., 2016; Szakács et al., 2020), determining the gene content of the introgressed QTLs would be highly beneficial. If the size of wheat–rye translocation chromosomes has changed significantly relative to the normal wheat chromosomes, and the distribution of FISH microsatellite probes on them is appropriate, the application of MutChromSeq pipeline using bivariate chromosome flow-sorting in combination with EMS mutagenesis (Sánchez-Martín et al., 2016) could be an effective option to identify agronomically important genes on the transferred alien chromosomes as demonstrated for cloning disease resistance genes (Holden et al., 2022; Yu et al., 2022).

We have to note, however, that molecular cytogenetic analysis cannot be eliminated from the characterization of wheat-alien hybrid progenies during the alien gene transfer. Our results indicate that the parallel analysis using cytogenetic methods and high-resolution marker systems could be the most optimal for the precise detection of intergenomic rearrangements. The 7RL-6RL chromosome arm is present in two kinds of translocation: 3RS.7RL-6RL and 7RS.7RL-6RL. As these rearranged rye chromosomes can be found as additions, the translocation between the arms 6RL and 7RL arose directly after the cross between *S. cereale* and *S. strictum* ssp. *anatolicum* or during the development of the cultivar Kriszta, rather than after the cross between Mv9kr1 and ‘Kriszta.’ This 6R/7R translocation is not unique. The studies by Benito et al. (1991) on the location of the endopeptidase-1 (*Ep-1*) gene on *S. cereale* and *S. montanum* (syn. *S. strictum*) chromosomes provided biochemical evidence that one of the three translocations evolutionary separating *S. cereale* from *S. montanum* (Singh and Röbbelen, 1977) had occurred between 6RL and 7RL. This suggests that the translocation in the genotypes 3RS.7RL-6RL and 7RS.7RL-6RL between the 6RL and 7RL arms may be of compensating type. The seed set data of these addition lines suggest that the long arm 7RL-6RL does not affect this trait but the short arm of the translocation chromosome does. It seems that 7RS has a strong yield-decreasing effect as the grain number per ear values for the 7RS.7RL-6RL disomic addition, as well as the 7RS ditelosomic addition, are extremely low (2 and 5 on average, respectively), while those for the 3RS.7RL-6RL addition line are the highest (37 grains on average) among the studied genotypes.

The line disomic for the 3RS isochromosome (carrying four copies of the 3RS chromosome arm) also shows good fertility, with an average of 34 grains per ear. Based on the grain number per ear value (24 on average), which is higher than that of the non-compensating translocation line T1BL.5RL (19 on average) but lower than that of the addition line 3RS.7RL-6RL, it is difficult to predict whether the translocation composed of a 3RS arm and a shorter wheat chromosome arm (segment) is compensating or not. The weak, terminal pSc119.2 signal suggests that the wheat arm is either 3DS or 2BS. Nevertheless, rye chromosomes pair more frequently with B-genome chromosomes than with those from the A and D genomes (Naranjo and Fernández-Rueda, 1996).

The existence of intergenomic relationships between the group-4 chromosomes of wheat and 7R, as well as group-7 chromosomes of wheat and 4R, has long been known (Zeller and Koller, 1981; Devos et al., 1993). So far, only two fertile translocation lines involving these chromosomes have been published. One of them is composed of 4A (or segment) and 7RS arms (Zeller and Koller, 1981), and the other one carries 7DL and *S. montanum* 4RL chromosome arms (Lukaszewski et al., 2001).

The present study focused on the detection of wheat–*S. cereanum* introgressions and the precise characterization of their genome structure. Because of the limited seed set, a detailed evaluation of agronomic performance, abiotic and biotic stress tolerance, and grain quality traits is not possible. However, several valuable traits have previously been mapped on chromosome 4R and chromosome arms 3RS, 5RL, 6RL, and 7RS, implying that the new wheat–*S. cereanum* introgression lines could be important gene sources for wheat improvement.

3RS harbors the stem rust resistance gene *Sr27* (Rao, 1979). Although translocation lines carrying 3RS have been developed (Marais and Marais, 1994), they have not been used in wheat breeding because of their “detrimental” effect on grain yield (Marais, 2001). In contrast, the Mv9kr1-’Kriszta’ addition line disomic for the 3RS isochromosome yields 34 ± 9.6 grains per ear, comparable to cultivated wheat. It might therefore be a realistic plan for the future to produce translocations with high yields. One of the most efficient aluminum tolerance genes found in the cultivated species of Triticeae, *Alt2* (Aniol and Gustafson, 1984), is also located on 3RS (Aniol, 2004).

The disomic addition of chromosome 4R increases the number of grains per spike (An et al., 2019), total protein and arabinoxylan content, and stripe rust resistance in wheat (Szakács et al., 2016). The 4RL chromosome arm carries a dominant locus (*Alt3*) for aluminum tolerance (Miftahudin et al., 2002) and resistance genes for powdery mildew (Duan et al., 2017; Ma et al., 2020).

A good copper efficiency in rye has been associated with 5RL (Schlegel et al., 1991). Dwarfing genes *ct2* and *Ddw1* (Plaschke et al., 1993; Korzun et al., 1996) and a stripe rust resistance gene (Xi et al., 2019) have also been mapped to this rye chromosome arm.

7RS carries the aluminum tolerance gene *Alt4* (Matos et al., 2005) and genes for zinc efficiency (Schlegel et al., 1999). Using CS-’Imperial’ disomic addition lines, it was shown that chromosome 7R increases the drought tolerance in wheat (Mohammadi et al., 2003), but the location on chromosome arms of the responsible genes is not known.

Several studies have found that chromosome arm 6RL is a potential gene source for wheat resistance breeding. On the 6RL arm of the rye cv. Prolific, a powdery mildew resistance gene (*Pm20*) was physically mapped by Friebe et al. (1994) to a distal position. Additional loci for resistance to powdery mildew on 6RL have been found in the rye cultivars Jingzhouheimai (Wang et al., 2010) and Kustro (Du et al., 2018). On this chromosome arm of rye cv. Merced, a novel stripe rust resistance gene (*Yr83*) has been reported recently (Li et al., 2020) and physically located at the fraction length 0.87–1.00, which corresponds to the 806–881-Mb region on the ‘Lo7’ reference genome (Li et al., 2022). Given that the small-sized 6RL fragment of the Mv9kr1-’Kriszta’ 3RS.7RL-6RL translocation chromosome is located terminally and the disomic addition line carrying this chromosome has good fertility, we consider this genetic material as a candidate for wheat resistance breeding. This is supported by field observations showing adult plant resistance of this genotype to naturally occurring stripe rust infections in years when the pathogen was present in the breeding nursery. It should be noted that detailed resistance tests in seedling and adult plant stages with monosporic isolates are needed to confirm the presence of an effective *Yr* resistance gene on this 6RL chromosome fragment. In addition, a translocation line containing only the short 6RL segment must be developed in order to capture the gene(s) of interest without the undesirable linkage drag caused by the 3RS.7RL chromosome part. Türkösi et al. (2022) introduced the *ph1b* mutation from Chinese Spring *ph1b* mutants into the winter wheat line Mv9kr1, which is the wheat genetic background of the BC_2_F_8_ generation. The Mv9kr1*ph1b* mutant line presents a promising tool for inducing the desired translocation. We also plan to convert the 1R–7R chromosome-specific SNPs to Kompetitive Allele-Specific PCR (KASP) markers for faster and less expensive screening of wheat lines for the presence of introgressed chromatin as it was demonstrated for *Ae. mutica* (King et al., 2017). This will enable us to identify additional introgression lines that were previously unidentified, such as those that carry 2R chromatin.

Great efforts are being made to increase the genetic diversity of wheat in order to improve its yield, stress tolerance, and disease resistance. Wheat–rye introgression lines offer promising genetic materials for achieving this goal, as cultivated and wild rye chromosomes harbor many useful genes hitherto unexploited in wheat breeding as it was systematically reviewed by Crespo-Herrera et al. (2017). The present study demonstrates that high-throughput DArTseq genotyping accurately identifies the “exotic” rye chromatin of *S. cereanum* in the wheat genetic background. However, molecular cytogenetic analyses (FISH, GISH) are still required to determine the precise chromosomal composition (monosomic, disomic, etc.) of potentially agronomically useful prebreeding populations. The genotypes from the wheat–*S. cereanum* BC_2_F_8_ generation, particularly the translocation (T1BL.5RL, Twheat.3RS) and addition (3RS isochomosomic, 4R, and 3RS.7RL-6RL) lines with good seed set, can be potentially valuable resources for breeding stress-tolerant or disease-resistant wheat cultivars. However, further experiments will be needed to prove if the rye chromosome arms introgressed from *S. cereanum* into wheat carry the effective alleles of the aforementioned genes. Future research will address this issue.

## Data Availability

The datasets presented in this study can be found in online repositories. The names of the repository/repositories and accession number(s) can be found in the article/**Supplementary Material**.

## Supplementary material

Supplementary Figure 1 Heatmap representation of SilicoDArT marker densities (obtained at IDT 93%) on each pseudomolecule of rye (1R-7R). Reads from the investigated genotypes were aligned to the ‘Lo7’ chromosome-scale assembly. The numbered scales show chromosome lengths in megabase pairs (Mbp), and the colored scales depict marker densities (number of markers per 10 Mbp).

Supplementary Figure 2 Heatmap representation of the SNP marker densities (obtained at IDT 93%) on each pseudomolecule of rye (1R-7R). Reads from the investigated genotypes were aligned to the ‘Lo7’ chromosome-scale assembly. The numbered scales show chromosome lengths in megabase pairs (Mbp), and the colored scales depict marker densities (number of markers per 10 Mbp).
