# Supplementary figures and images for: DArTseq genotyping facilitates the transfer of “exotic” chromatin from a Secale cereale × S. strictum hybrid into wheat
Source: Front Plant Sci. 2024 Sep 6;15:1407840. doi: 10.3389/fpls.2024.1407840 (PMC11412823; doi:10.3389/fpls.2024.1407840)

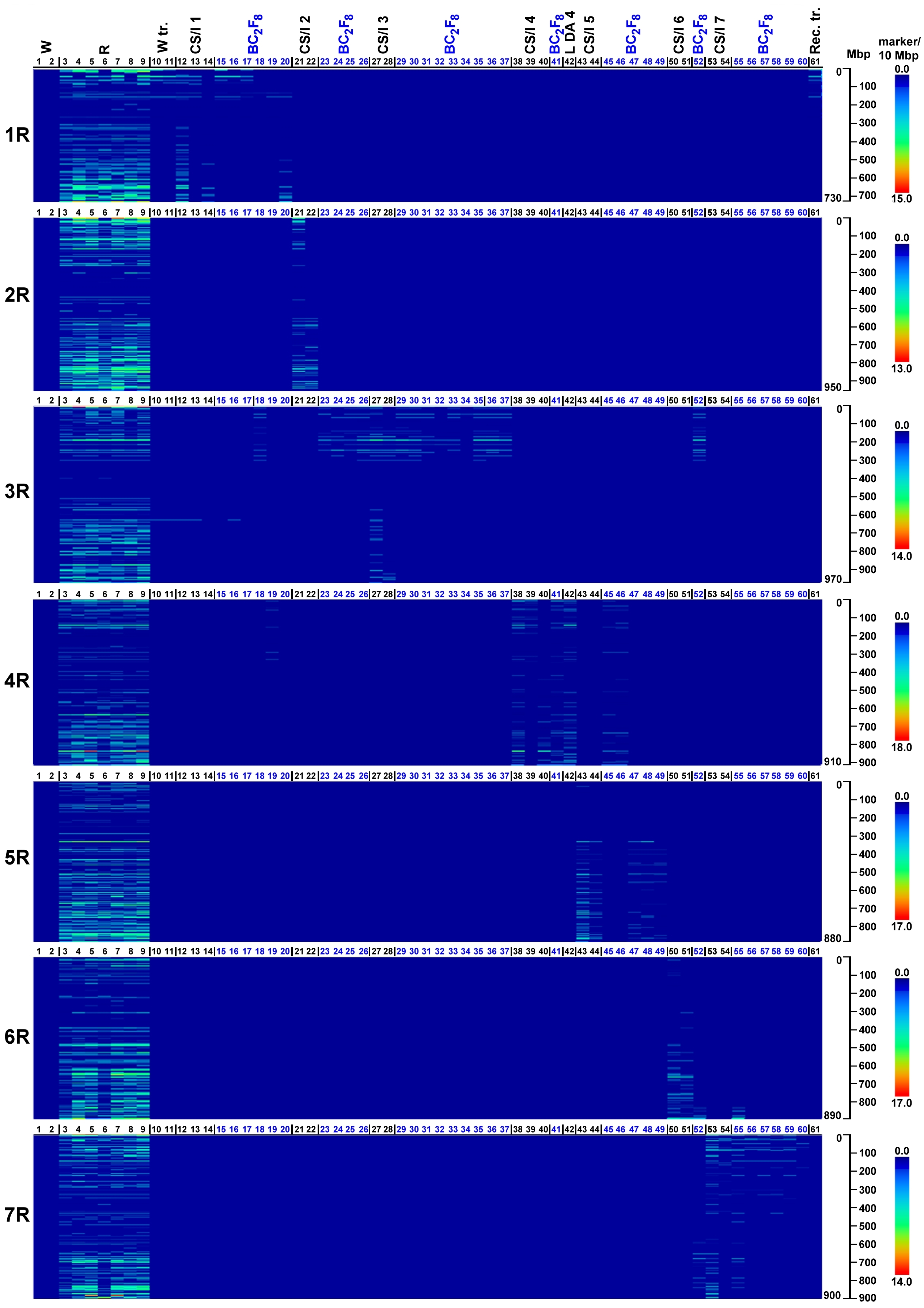

Supplement: Supplementary Figure 1 — Heatmap representation of SilicoDArT marker densities (obtained at IDT 93%) on each pseudomolecule of rye (1R-7R). Reads from the investigated genotypes were aligned to the ‘Lo7’ chromosome-scale assembly. The numbered scales show chromosome lengths in megabase pairs (Mbp), and the colored scales depict marker densities (number of markers per 10 Mbp). [file Image1.jpeg]

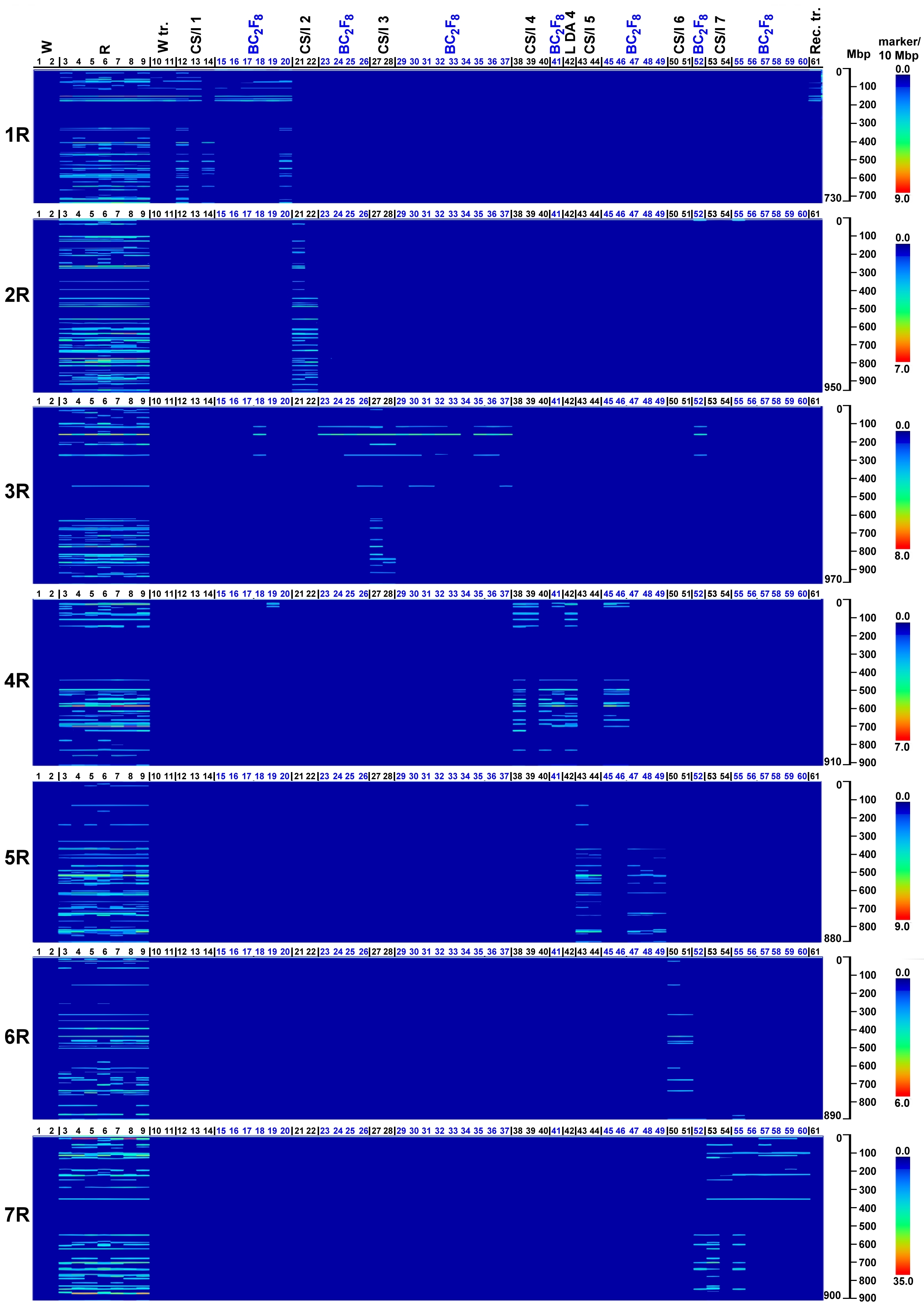

Supplement: Supplementary Figure 2 — Heatmap representation of the SNP marker densities (obtained at IDT 93%) on each pseudomolecule of rye (1R-7R). Reads from the investigated genotypes were aligned to the ‘Lo7’ chromosome-scale assembly. The numbered scales show chromosome lengths in megabase pairs (Mbp), and the colored scales depict marker densities (number of markers per 10 Mbp). [file Image2.jpeg]
